# Metformin Downregulates PD-L1 Expression in Esophageal Squamous Cell Carcinoma by Inhibiting IL-6 Signaling Pathway

**DOI:** 10.3389/fonc.2021.762523

**Published:** 2021-11-22

**Authors:** Yao Lu, Dao Xin, Lulu Guan, Mengli Xu, Yalan Yang, Yu Chen, Yuanyuan Yang, Andrea Wang-Gillam, Li Wang, Shanggang Zong, Feng Wang

**Affiliations:** ^1^ Department of Oncology, The First Affiliated Hospital of Zhengzhou University, Zhengzhou, China; ^2^ Department of Oncology, Washington University School of Medicine, St. Louis, MO, United States; ^3^ Henan Academy of Medical Sciences, Zhengzhou, China

**Keywords:** metformin, PD-L1, anti-PD-1 antibody, esophageal squamous cell carcinoma, IL-6/JAK2/STAT3 signaling pathway

## Abstract

**Purpose:**

To characterize the mechanism by which metformin inhibits PD-L1 expression in esophageal squamous cell carcinoma (ESCC) and to evaluate the effect of metformin on the antitumor immune response.

**Methods:**

The Cancer Genome Atlas (TCGA) database was used to analyze the correlations between IL-6 and prognosis and between IL-6 and PD-L1 gene expression in esophageal cancer. Reverse transcription-quantitative polymerase chain reaction (RT-PCR), Western blotting and immunofluorescence were used to study the mechanism by which metformin affects PD-L1 expression. Additionally, T cell function was assessed in a coculture system containing ESCC cells and peripheral blood mononuclear cells (PBMCs) treated with metformin or IL-6. In an *in vivo* assay, we used a model established with NPIdKO™ mice, which have a reconstituted immune system generated by transplanting PBMCs through intravenous injection, to evaluate the effect of metformin on tumors.

**Results:**

The TCGA esophageal cancer data showed that IL-6 expression was positively correlated with PD-L1 expression and that patients with high IL-6 expression had a significantly lower overall survival rate than patients with low IL-6 expression. PD-L1 expression in ESCC cell lines was significantly inhibited by metformin *via* the IL-6/JAK2/STAT3 signaling pathway but was not correlated with the canonical AMPK pathway. In the coculture system, the metformin pretreatment group showed higher T cell activation and better T cell killing function than the control group. Animal experiments confirmed that metformin downregulated PD-L1 expression and that combination treatment with metformin and PD-1 inhibitors synergistically enhanced the antitumor response.

**Conclusions:**

Metformin downregulated PD-L1 expression by blocking the IL-6/JAK2/STAT3 signaling pathway in ESCC, which enhanced the antitumor immune response.

## Introduction

Metformin is a widely used medication that has been prescribed to treat type 2 diabetes for decades (1). In recent years, metformin has been found to improve the survival prognosis of cancer patients in the clinic (2, 3). One study also showed that the routine dosage of metformin can exert anticancer effects (4). An increasing number of experimental studies have shown that metformin can effectively suppress tumor cell proliferation and increase chemosensitivity with synergistic effects (5, 6). The combination of metformin with classic chemotherapeutic agents, including paclitaxel or cisplatin, could significantly suppress tumor cell growth and prolong remission in a xenograft model. It has also been reported that metformin can overcome tyrosine kinase inhibitor (TKI) resistance by reversing epithelial-mesenchymal transition (EMT) and inhibiting IL-6 signaling pathways. Metformin in combination with gefitinib significantly enhances the efficacy of targeted therapy in non-small cell lung cancer (NSCLC) (7).

Esophageal cancer is the sixth most prevalent cancer worldwide. The majority of the global burden of esophageal cancer remains esophageal squamous cell carcinoma (ESCC), and most ESCC cases occur in China (8, 9). Although the incidence and mortality of ESCC have gradually declined during the last three decades, the dismal prognosis of locally advanced or metastatic ESCC is still threatening public health (10). Recently, immune checkpoint inhibitors (ICIs) and monoclonal antibodies against immune checkpoint molecules, such as anti-CTLA-4, anti-PD-1 and anti-PD-L1, have been studied in various types of cancers, and these agents are routinely used in the clinic (11, 12). In ESCC, antibodies targeting PD-1/PD-L1 have produced promising results (13). Pembrolizumab and nivolumab are anti-PD-1 ICIs, whereas atezolizumab is an anti-PD-L1 ICI. These ICIs have been used in combination with different chemotherapies in prospective phase II and III clinical trials as well as in retrospective studies (14–16).

Various and complex factors are responsible for inducing the expression of PD-L1, which plays a key role in the tumor microenvironment (TME) (17). It has been reported that metformin has a role in adjusting the TME, including promoting antitumor immunity *via* the endoplasmic reticulum-associated degradation of PD-L1 and induction of CD39 and CD73 expression reductions to block myeloid-derived suppressor cell activity in breast and ovarian cancer (18). More interestingly, patients with NSCLC receiving concurrent metformin and anti-PD-1 therapy have a higher response rate and longer time to progression (TTP) than patients treated with the anti-PD-1 antibody alone (19). The results of a phase II clinical trial showed that low-dose metformin reprogrammed the tumor immune microenvironment in human esophageal cancer (20). However, for ESCC, the molecular mechanism by which metformin regulates PD-L1 expression is largely unknown.

The purpose of this study was to characterize the mechanism by which metformin inhibits PD-L1 expression in ESCC and to evaluate the effect of metformin on the antitumor immune response.

## Materials and Methods

### Cell Lines and Cell Culture

The human ESCC cell lines KYSE-450 and TE-7 were purchased from the Type Culture Collection of the Chinese Academy of Sciences (Shanghai, China). All the cell lines were cultured in RPMI 1640 medium supplemented with 10% fetal bovine serum (FBS) and antibiotics (10,000 U/ml penicillin and 10 μg/ml streptomycin). All cells were maintained in a humidified incubator at 37°C with 5% carbon dioxide. Drugs, including metformin, IL-6, Stattic and Compound C, were purchased from MedChemExpress (MCE).

### Plasmid Construction and Transfection

shRNA constructed to target JAK2 was inserted into the GV493 vector provided by GenePharma (Shanghai, China) (**Supplementary Table 1**). All plasmids were added with pHelper 1.0 and pHelper 2.0 plasmids into the supernatant of revived 293T cell culture (**Supplementary Figure 1**). Growth medium was removed after 24 hours of incubation, and fresh DMEM-high glucose (Sigma, USA) with 10% FBS (Dakewe, New Zealand) was added. Then, 293T cells were cultured for another 48 hours. The corresponding lentiviruses were obtained after ultracentrifugation of the collected supernatant for 4 hours. KYSE450 cells were transfected with virus cloned with a stable JAK2 knockdown sequence. The above cells were then purified with 1.5 µg/mL puromycin (Solarbio) for 2 weeks.

### Western Blot Analysis

Cells were harvested and suspended in RIPA lysis buffer (Thermo Scientific™, 89901) containing a protease inhibitor cocktail (Thermo Scientific™, 78437) and the phosphatase inhibitor cocktail PhosSTOP (Cell Signaling Technology, 5872). After incubation on ice for 30 minutes, the cell lysate was centrifuged at 12,000 rpm for 20 minutes at 4°C. The protein content of the supernatant was quantified using Thermo Pierce™ BCA Protein Assay Reagent (Thermo Scientific™, 23228). A total of 30 μg protein per well was separated by 10% sodium dodecyl sulfate–polyacrylamide gel electrophoresis and transferred to polyvinylidene difluoride membranes (Thermo Scientific™, 0.2 μm, LC2002). The membranes were immunoblotted with primary antibodies against PD-L1 (Cell Signaling Technology, 13684), phospho-JAK2 (Tyr1007/1008, Wanlei, WL02997), JAK2 (Protein Group, Wuhan, 17670-1-AP), phospho-STAT3 (Y705, Abcam, 76315), STAT3 (Protein Group, Wuhan, 10253-2-AP), phospho-ERK1/2 (Thr202/Tyr204, Cell Signaling Technology, 4370), ERK1/2 (Abcam, 17942), phospho-AMPK (Thr172, Abcam, 131357), AMPK (Protein Group, Wuhan, 66536-1-Ig), phospho-ACC (Ser473, Cell Signaling Technology, 3661), and ACC (Protein Group, Wuhan, 67373-1-Ig) overnight at 4°C. After washing three times, the membranes were further incubated with horseradish peroxidase-conjugated goat anti-rabbit (Abcam, 150077) or goat anti-mouse (Abcam, 150117) secondary antibodies (1:2000, Santa Cruz, CA) at room temperature for 2 hours. Signals were detected by Supersignal West Dura Luminol/Enhancer solution.

### RNA Extraction and PCR

To quantify PD-L1 mRNA expression, total RNA was extracted from cells with TRIzol reagent (Invitrogen), and cDNA was synthesized using TaqMan MultiScribe Reverse Transcriptase (SuperScript™ IV Reverse Transcriptase, Thermo Fish) according to the manufacturer’s instructions. Quantitative real-time PCR (RT-PCR) analysis was performed using an ABI Prism 7900-HT Sequence Detection System (96-well, Applied Biosystems). For RT-PCR, the following primers were used for amplification: PD-L1, forward primer 5′-TGGCATTTGCTGAACGCATTT-3′ and reverse primer 5′-TGCAGCCAGGTCTAATTGTTTT-3′; and GAPDH, 5′-GGAGCGAGATCCCTCCAAAAT-3′ (forward) and 5′-GGCTGTTGTCATACTTCTCATGG-3′ (reverse). The experiments were performed in triplicate. The relative expression of PD-L1 was normalized to GAPDH expression.

### Staining of Tissue PD-L1

Immunohistochemical analysis of PD-L1 in 4-μm paraffin-embedded archived ESCC tissue biopsy specimens was performed in accordance with a standard protocol using commercial antibodies. After deparaffinization and dehydration, sections were placed in 10 mM sodium citrate buffer (pH = 6.0), autoclaved at 121°C for 10 minutes, and then incubated in normal goat serum (KL-D1418; KALANG, Zhengzhou, China) for 30 minutes to block nonspecific antibody binding sites. Each section was then incubated for 10 minutes at room temperature with an anti-PD-L1 rabbit monoclonal antibody (Abcam, 228415) diluted 1:500 in phosphate-buffered saline (PBS) containing 1% bovine serum albumin. PBS was used as a negative control.

### Enzyme-Linked Immunosorbent Array (ELISA)

The Human IL-6 ELISA Kit and Human IL-2 ELISA Kit were purchased from Protein Group, Wuhan. The levels of IL-6 and IL-2 in tissue lysates were measured according to the manufacturers’ protocols. Absorbance at 450 nm was measured using a microplate reader (Bio-Rad, Hercules, CA, USA).

### T Cell Activation Assay and T Cell-Mediated Tumor Cell-Killing Assay

First, human peripheral blood mononuclear cells (PBMCs) were isolated from peripheral blood donated by healthy volunteers by Ficoll-Paque density centrifugation. KYSE-450 cells were treated with metformin, IL-6 or PBS for 24 hours. PBMCs were cultured in RPMI medium containing 10% FBS and phytohemagglutinin (PHA) (Sigma) at 0.1 μg/mL as indicated for 3 days, followed by stimulation with anti-CD3/CD28 antibodies (100 ng/mL) to activate T cells (PBMCs). Then, the activated PBMCs were added to the coculture system containing KYSE-450 cells at a ratio of 2:1. After 24 hours of coculture, the wells were washed with PBS 3 times to remove T cells. The T cells were then collected and lysed. ERK phosphorylation was evaluated by Western blotting. The cell-free supernatant from the coculture system was collected by centrifugation at 12,000 rpm for IL-2 analysis by ELISA.

T cells (PBMCs) were activated with ImmunoCult™ Human CD3/CD28 T Cell Activator (100 ng/mL; STEMCELL Technologies, 19071) and IL-2 (10 ng/mL; Abcam, 174444). After 4 days of coculture of tumor cells and T cells with metformin, IL-6 or PBS in 12-well plates, the wells were washed with PBS 3 times to remove the T cells. The surviving tumor cells were fixed and stained with a crystal violet solution (Sigma, V5265). The dried plates were scanned, stained, solubilized in 100 μL 33% glacial acetic acid, and shaken until the color was uniform. The absorbance at 450 nm was measured using a spectrometer.

### Animal Study

Male NPIdKO™ mice (NOD-Prkdc^scid^-Il2^rgem1IDMO^) (6 weeks old) were purchased from Beijing IDMO Company, and all animal experimental procedures were conducted with the approval of the Animal Ethics Committee of The First Affiliated Hospital of Zhengzhou University. NPIdKO™ mice are immunodeficient with NPI™ as the genetic background, resulting from the knockout of the MHC I molecule β2-microglobulin (β2M) gene and MHC II molecule IAβ gene. These mice lack mature T, B and natural killer (NK) cells, produce no immunoglobulin; and have dendritic cells (DCs) with abnormal functions.

PBMCs from healthy donors were diluted to a ratio of 1:2 with PBS, and mononuclear cells were isolated by using density-gradient centrifugation with Ficoll medium. Approximately 1 × 10^5^ freshly isolated PBMCs were injected intravenously into NPIdKO™ mice. Then, the mice were randomized into four treatment groups in all of the experiments (each group contained 5 animals).

The TE-7-luc cell line (TE-7 cells labeled with luciferase) was kindly provided by Dr. Ruizhe Li (Department of Pathology, The First Affiliated Hospital of Zhengzhou University, China). Fifteen days after PBMCs were injected, TE-7-luc cells (1 × 10^6^ cells) in 100 μl medium were subcutaneously administered into the right flank of NPIdKO™ mice.

Treatments began when the tumor size was 100 mm^3^. The mice in different groups were administered metformin (35 mg/kg/d, diluted in 0.1 mL normal saline), anti-PD-1 (camrelizumab, Hengrui, China) monoclonal antibodies (200 μg/3 days/mouse, diluted in 0.1 mL normal saline), combination treatment with metformin and the anti-PD-1 antibodies, or control treatment (0.1 mL normal saline/day).

Tumor size was measured every 3 days for 4 weeks with a caliper, and tumor volume was calculated using the following formula: width × width × length/2. Tumor volume was also measured by quantifying bioluminescence intensity with a small-animal *in vivo* imaging system (IVIS 200; Caliper Life Sciences). At the endpoint of experiments, defined as whichever came first among a tumor volume greater than 1,000 mm^3^, tumor ulceration, or study end, the mice were euthanized, and the tumors were harvested and weighed quickly. Then, the tumors were fixed in neutral buffered formalin and embedded in paraffin for immunohistochemical analyses.

### The Cancer Genome Atlas (TCGA) Database Collection and Analysis

The Cancer Genome Atlas- Esophageal Cancer (TCGA-EC) RNAseqV2 gene expression data and clinical data were obtained from the TCGA. Data Portal Transcriptional values were Log2-transformed from the normalized fragments per kilobase transcript per million mapped reads values using R package “limma” in R 3.6.0. We divided patients into different groups by the medians of IL-6 genes expression. Kaplan-Meier analysis was performed for survival curves and the significance was determined by log-rank, which was completed by R software. The associations between IL-6 levels and clinicopathologic characteristics of patients were analyzed using one-way analysis of variance (ANOVA). The correlation between IL-6 and PD-L1 levels was analyzed using the Pearson correlation coefficient.

### Statistical Analysis

Representative results from three independent experiments are shown in the present study. Numerical data are presented as the mean ± standard deviation of the mean (SD). The p-values between two experimental groups were determined by a two-tailed Student’s t test, and p-values less than 0.05 were considered significant.

## Results

### TCGA Data Showed That IL-6 Expression Was Positively Correlated With PD-L1 Expression in Esophageal Cancer Tissues

**Figure 1:**
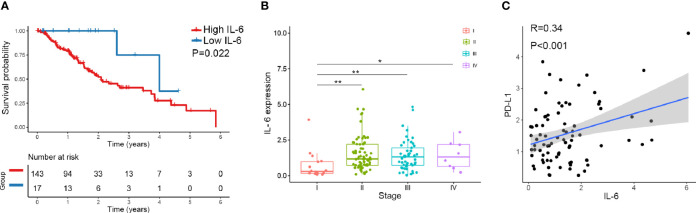
The expression of IL-6 and PD-L1 in esophageal cancer tissues was analyzed by TCGA database. **(A)** The overall survival probability of patients with high IL-6 expression was significantly lower than that of patients with low IL-6 expression. **(B)** IL-6 expression was significantly decreased in low-stage tissues compared with higher-stage tissues. **(C)** PD-L1 was positively correlated with IL-6 expression in esophageal cancer. *p < 0.05, **p < 0.01.

We downloaded IL-6 and PD-L1 gene expression information and the corresponding clinical data for esophageal cancer from TCGA database (https://cancergenome.nih.gov). Altogether, 160 esophageal cancers from TCGA with normalized gene expression and specific clinical status were collected and analyzed. For the transcriptional data for IL-6 in esophageal cancer tissue, the median value was selected as the cutoff point, and the patients were divided into high and low IL-6 expression groups. The results showed that high expression of IL-6 was associated with a poor prognosis (P=0.022) (**Figure 1A**). Combined with tumor stage data, the results suggested that IL-6 expression was significantly decreased in low-stage tissues compared with higher-stage tissues (**Figure 1B**). Furthermore, the correlation between IL-6 and PD-L1 gene expression was evaluated, and a positive correlation was found between PD-L1 and IL-6 expression in esophageal cancer (R=0.34, P<0.01) (**Figure 1C**).

### Metformin Suppresses the IL-6 Signaling Pathway and Downregulates the Expression of PD-L1 *In Vitro*

**Figure 2:**
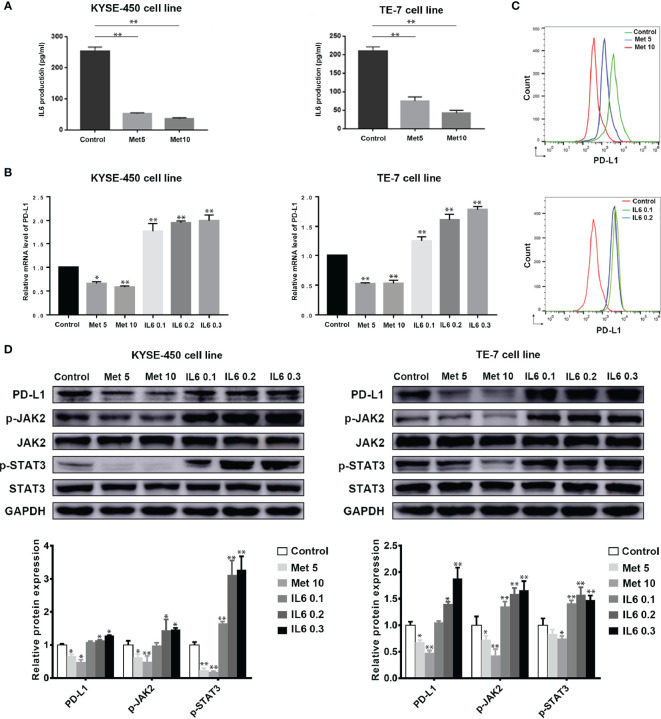
Metformin suppresses the IL6 signaling pathway and downregulates the expression of PD-L1 *in vitro*. KYSE-450 and TE-7 cells were treated with increasing concentrations of metformin (5 to 10 mM), IL-6 (0.1 to 0.3 ng/ml) or PBS for 36 (h) **(A)** The expression of IL-6 in each group was detected by ELISA. **(B)** mRNA expression levels of PD-L1 in cells treated with metformin or IL-6 were analyzed by RT-PCR. **(C)** The expression of PD-L1 by flow- cytometric analysis after metformin or IL-6 treatment. **(D)** Western blot analysis and quantification of PD-L1, phosphorylated- Jak2 (p-Jak2), Jak2, phosphorylated- Stat3 (p-Stat3), Stat3, and GAPDH were performed. Error bars represent the standard deviation. *p < 0.05, **p < 0.01.

To investigate the role of metformin in esophageal cancer, we compared the antitumor effects of metformin on ESCC cell lines, including KYSE-450 and TE-7. Interestingly, we found that metformin treatment significantly decreased the expression of IL-6 (**Figure 2A**). Based on the related results for the TCGA data, we next examined the effect of metformin on PD-L1 and found that metformin could significantly inhibit the expression of PD-L1 in a dose-dependent manner. The effect could be reversed by adding exogenous IL-6, which suggests that the downregulation of PD-L1 induced by metformin is associated with the IL-6 signaling pathway (**Figures 2B, C** and **Supplementary Figure 2A**). The involvement of the JAK2/STAT3 signaling pathway in IL-6 signaling has been shown by many results (7, 21, 22). In our study, metformin alone effectively downregulated JAK2 and STAT3 activation and the PD-L1 expression level in the two cell lines. To expand on this result, we also examined the role of IL-6 in PD-L1 expression and found that IL-6 significantly activated the JAK2/STAT3 signaling pathway and increased the level of PD-L1 expression (**Figure 2D**). Therefore, we speculated that the reduction in PD-L1 expression induced by metformin was related to the IL-6/JAK2/STAT3 signaling pathway.

### The Reduction in PD-L1 Expression Induced by Metformin Depends on the IL-6/JAK2/STAT3 Signaling Pathway

**Figure 3:**
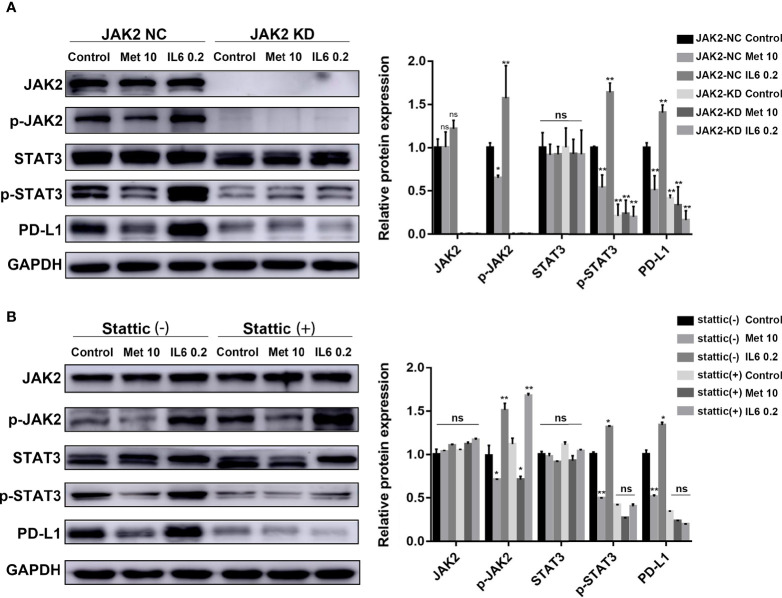
The reduction in PD-L1 expression induced by metformin depends on the IL6/JAK2/STAT3 signaling pathway. **(A)** KYSE-450 (JAK2 NC) and KYSE450 (JAK2 KD) cells were treated with metformin (10 mM), IL-6 (0.2 ng/ml) or PBS for 36 (h). **(B)** KYSE-450 cells were pretreated with the STAT3 phosphorylation inhibitor Stattic (5 μM) for 2 h and then treated with metformin (10 mM), IL-6 (0.2 ng/ml) or PBS for 36 (h). Western blot analysis and quantification of phosphorylated- Jak2 (p-Jak2), Jak2, phosphorylated- Stat3 (p-Stat3), Stat3, PD-L1, and GAPDH were performed. Error bars represent the standard deviation. *p < 0.05, **p < 0.01; ns, no significance.

A mutant KYSE450 (JAK2 KD) cell line was prepared by knocking out the JAK2 gene in KYSE-450 cells (**Supplementary Figure 2B, C**). The expression of p-STAT3 and PD-L1 in the KYSE450 (JAK2 KD) cell line was significantly reduced compared with that in the KYSE450 (JAK2-NC) cell line. The regulatory effects of metformin and IL-6 on PD-L1 disappeared when the JAK2 gene was knocked down (**Figure 3A**).

To further verify that p-STAT3 can regulate the expression of PD-L1, we pretreated KYSE450 cells with Stattic, which could inhibit STAT3 phosphorylation. The regulatory effects of metformin and IL-6 on PD-L1 disappeared when STAT3 phosphorylation was inhibited (**Figure 3B**). In brief, metformin downregulated PD-L1 expression through the JAK2/STAT3 signaling pathway.

### Metformin Attenuates IL-6 Downstream Signaling Through an AMPK-Independent Pathway

**Figure 4:**
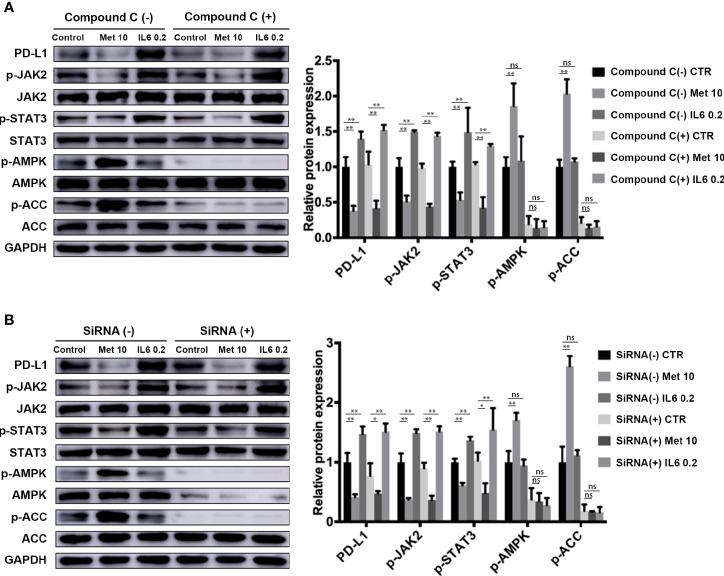
Metformin attenuates IL-6 downstream signaling through an AMPK-independent pathway. **(A)** KYSE-450 cells were pretreated with the AMPK inhibitor compound C (5 μM) for 4 h. **(B)** Negative control siRNA and AMPKα 1/2 siRNA (80 nM) were transfected into KYSE-450 cells for 48 h. Cells were treated with metformin (10 mM) or IL-6 (0.2 ng/mL) for 36 h, independently. Western blot analysis and quantification of p-Jak2, Jak2, p-Stat3, Stat3, p-AMPK, AMPK, p-ACC, ACC and GAPDH were performed. Data are mean ± SEM. Kruskal–Wallis ANOVA combined with *post- hoc* Dunn’s multiple comparison test (two tailed) was performed. *p < 0.05, **p < 0.01; ns, no significance.

Metformin is a well-known AMP-activated protein kinase (AMPK) activator. In our study, we investigated whether the AMPK signaling pathway is involved in the downregulation of PD-L1 expression induced by metformin. The levels of phosphorylated AMPK and acetyl-CoA carboxylase (ACC), an AMPK target, were both decreased in the AMPK inhibitor Compound C pretreatment group (**Figure 4A**) and AMPKα 1/2-specific siRNA-transfected group (**Figure 4B**), demonstrating the loss of AMPK activity. Notably, metformin still decreased PD-L1 expression by inhibiting JAK2/STAT3 signaling. This result indicated that metformin downregulated PD-L1 expression in a manner independent of the AMPK pathway.

### Metformin Increases T Cell Activity Through the IL-6/PD-L1 Axis

**Figure 5:**
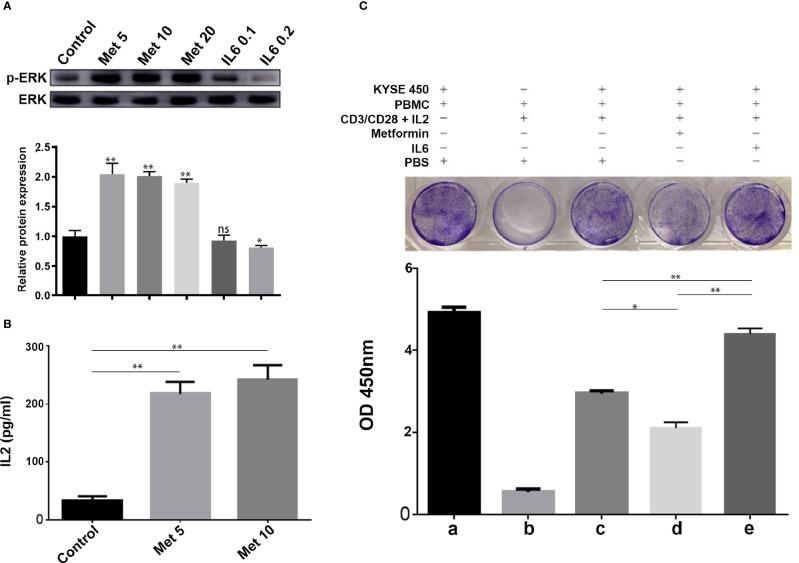
Metformin increases T cell activity through the IL-6/PD-L1 axis. **(A)** ERK phosphorylation was determined by Western blotting (quantification of p-ERK expression level shown at the bottom. **(B)** IL-2 secretion was measured using a human IL-2 ELISA kit. **(C)** T-cell-meditated tumor cell-killing assay in KYSE-450 cells incubated with metformin, IL6, or PBS, and a-e were the quantitative results of the above grouping. Data represent the mean ± SD of three independent experiments. *p < 0.05, **p < 0.01, ns, no significance.

It is well known that PD-L1 in tumor cells can lead to inactivation of T cells. First, human PBMCs were isolated from peripheral blood donated by healthy volunteers by Ficoll-Paque density centrifugation. Then, PBMCs were added to a coculture system containing KYSE-450 cells at a ratio of 2:1. To evaluate whether the downregulation of PD-L1 mediated by metformin could promote the CD3/CD28-triggered T cell activation signaling pathway, we generated T cell blasts by treating PBMCs with PHA to induce PD-1 expression. The results showed that metformin markedly promoted CD3/CD28-induced ERK phosphorylation in a dose-dependent manner (**Figure 5A**). The coculture supernatant was collected for IL-2 measurement. We found that IL-2 was markedly increased in the supernatant of the KYSE450/PBMC coculture system after metformin treatment (**Figure 5B**). Moreover, IL-6 inhibited CD3/CD28-induced ERK phosphorylation and PHA-induced IL-2 secretion, both of which are indicators of T cell activation. Next, we performed a T cell-mediated tumor cell-killing assay to examine the influence on T cell function. The results showed that metformin-induced PD-L1 downregulation protected the cytotoxic function of T cells, while this effect was reversed by IL-6 (**Figure 5C**).

### Metformin Effectively Suppresses Esophageal Tumor Growth *In Vivo*

**Figure 6:**
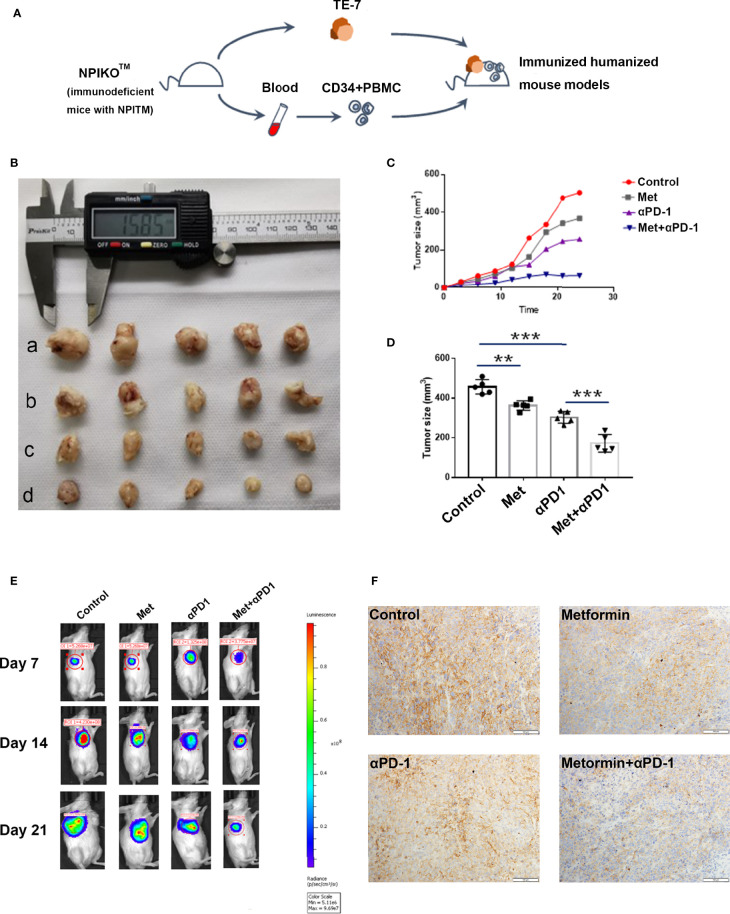
Metformin effectively suppresses esophageal tumor growth *in vivo*. **(A)** Construction of an immunohumanized mouse model. Tumor growth of ESCC cells (TE-7) in NPI mice was injected with metformin, anti-PD1 antibody or their combination as indicated. **(B)** Macroscopic appearance of the tumors Metformin effectively suppresses esophageal tumor growth *in vivo*. **(A)** Construction of an immunohumanized mouse model. Tumor growth of ESCC cells (TE-7) in NPI mice was injected with metformin, anti-PD1 antibody or their combination as indicated. **(B)** Macroscopic appearance of the tumors at 3 weeks after treatment with drugs. **(C)** The average tumor volumes were measured at the indicated time points (n=5 mice per group). **(D)** The tumor volumes at endpoint. **(E)** Small-animal *in vivo* imaging system indicating tumor growth at day 7, day 14 and day 21. **(F)** Immunohistochemistry showed the expression of PD-L1 in different groups. **p < 0.01, ***p < 0.001.

NPIdKO™ mice were subcutaneously implanted with TE-7 cells, followed by administration of metformin and an anti-PD-1 antibody as a monotherapy or combination therapy (**Figure 6A**). The anti-PD-1 antibody or metformin alone exhibited anticancer activity, and metformin significantly enhanced the efficacy of combination therapy in TE-7 cell-harboring mice, which was reflected by a tumor size assay and small-animal *in vivo* imaging system (**Figures 6B–E**). Consistent with the aforementioned mechanistic findings, immunohistochemical staining of tumor tissues showed that metformin reduced PD-L1 levels. In addition, metformin and anti-PD-1 combination therapy produced a significant reduction in the tumor expression of PD-L1 (**Figure 6F**). In this study, we did not observe any abnormalities in mouse body weight or behavior.

## Discussion

Several epidemiological studies have indicated that metformin treatment can lower the risks of several types of cancer in patients with diabetes. Patients with diabetes with breast cancer receiving metformin and neoadjuvant chemotherapy were shown to have a higher pathologic complete response rate than those who did not receive metformin (23). In mouse xenografts, metformin exerted comparable tumor-regressing effects when it was combined with a 4-fold reduced dose of doxorubicin, which is not effective as a monotherapy (24). Metformin inhibited the proliferation of NSCLC and breast cancer cell lines and blocked transformation in an inducible model system (25). These reports, together with our findings that metformin significantly enhances the effect of antitumor immunity on ESCC *in vitro* and *in vivo*, suggest that metformin has promising potential for use as a novel anticancer agent.

Although there are few esophageal cancer data in TCGA database, the current analysis indicates that high expression of IL-6 is associated with a poor prognosis. IL-6 exerts its effects by binding to a cell-surface type I cytokine receptor complex consisting IL-6R (CD126) and a common cytokine receptor signal-transducing subunit gp130, which forms a complex to activate STAT3 with the phosphorylation of Tyr705 *via* the JAK signaling pathway (26, 27). The activation of IL-6/JAK2/STAT3 signaling pathway plays an active role in tumor growth and progression (28–30). Some studies have shown metformin could inhibit IL-6 secretion and decrease IL-6 signaling activation in tumor cells (21, 31). A recent study found another novel mechanism for metformin to block the IL-6 signaling pathway by decreasing IL-6R expression on multiple myeloma cells (32). Our study observed metformin treatment significantly decreased the secretion of IL-6, and downregulated JAK2 and STAT3 activation in ESCC cells. This is a new exploration of anti-tumor mechanism of metformin in ESCC.

Some studies have suggested that activation of STAT3 signaling pathway regulates PD-L1 expression in ESCC (33–35). JAK2/STAT3 pathway, which is one of the best understood signal transduction cascades, plays a key role in tumor immune microenvironment (36). Abnormal activation of epidermal growth factor receptor (EGFR) is common in different types of cancer including ESCC, which can upregulates the expression of PD-L1 through STAT3 signaling pathway (34, 37). Silencing STAT3 and PD-L1 antibody injection in combination increased apoptosis in tumor cells and thus offers better anti-cancer activity (38). Our study also revealed that PD-L1 expression was association with IL-6/JAK2/STAT3 signaling pathway in ESCC. Recent studies show that PD-L1 is mainly regulated by JAK1 and JAK2, several STATs, and other modulators of the pathway and converged on the binding of IRF1 to the PD-L1 promoter (39). Other studies also demonstrate that STAT3 can directly act on the PD-L1 promoter to activate its transcription (40–42). These studies provide a reasonable explanation for the regulation of PD-L1 expression by STAT3.

Metformin has been shown to directly induce antitumor effects by inhibiting the PI3K-Akt/mTOR and Ras-MAPK signaling pathways critical for cancer progression and indirectly by systemically reducing glucose and insulin metabolism to attenuate cancer cell growth (43, 44). In addition, studies have recently shown that metformin regulates the differentiation and activity of T cells through an intrinsic pathway, suggesting that the antitumor effects of metformin may also be linked to the immune response (45). As an important immune checkpoint, PD-L1 can inhibit the activation of T cells and help tumor cells escape the recognition and killing of immune system. A previous study demonstrated that metformin could alter the PD-L1 glycan structure, promoting PD-L1 degradation and subsequently blocking immune-inhibitory signaling in breast cancer (46). A recent clinical trial showed that metformin can shift the tumor immune microenvironment from a protumoral state toward a more antitumoral state and reduce the expression of PD-L1 in ESCC (20). However, there is no clear molecular mechanism through which metformin modulates PD-L1 pathway in ESCC. Here, our results demonstrated a new regulatory mechanism for PD-L1 expression in ESCC.

In this study, we observed relatively robust activation of the IL-6 signaling pathway in ESCC cells, accompanied by increased phosphorylation of JAK2 and STAT3. Furthermore, adding IL-6 to a coculture system containing an ESCC cell line and PBMCs decreased the activation of T cells, abolished the killing function of these cells and upregulated PD-L1 expression in ESCC by reactivating the IL-6 signaling pathway. In this study, we found for the first time metformin downregulates PD-L1 expression by blocking the IL-6/JAK2/STAT3 signaling pathway, thereby enhancing the antitumor immune response. Our study further elucidated the mechanism by which metformin regulates the tumor immune microenvironment and provided a new idea for combined immunotherapy, including metformin.

Inhibitory immune checkpoint blockade by anti-CTLA-4, anti-PD-1, or anti-PD-L1 antibodies has provided substantial benefits to advanced cancer patients. Various approaches are being explored to expand the benefits and improve the efficacy of these ICIs, including ongoing clinical trials to evaluate the effects of combined blockade of CTLA4 and PD-L1 or PD-1 (47). In this regard, our findings indicated that metformin significantly improved the antitumor effects by downregulating PD-L1 without detectable toxicity and suggested that metformin has strong potential to be used in combination with immunotherapy.

## Conclusions

**Figure 7:**
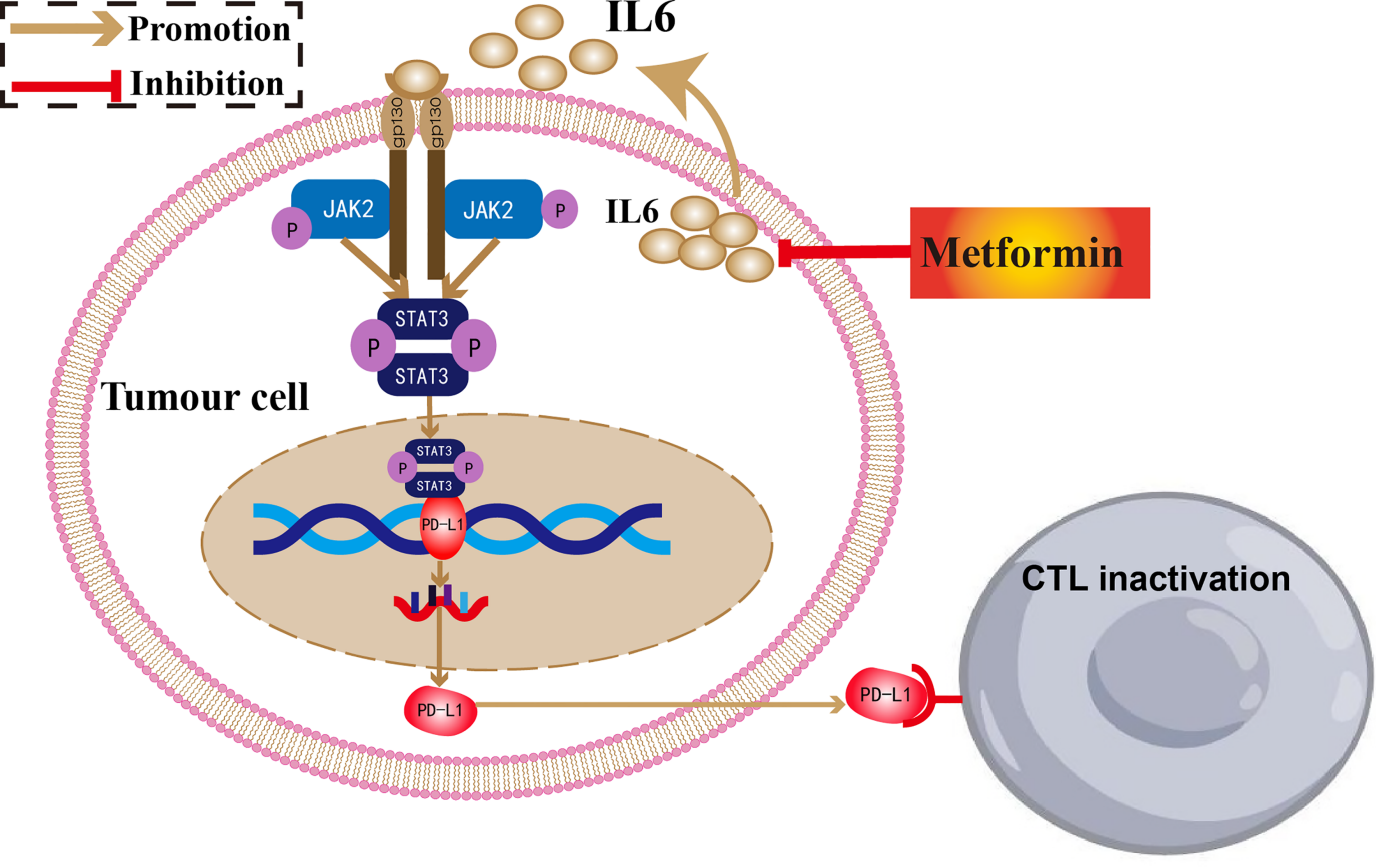
The antitumor mechanism of metformin in ESCC.

In conclusion, we demonstrated that metformin could inhibit PD-L1 expression and enhance T cell activation through the IL-6/JAK2/STAT3 signaling pathway. Combination treatment with metformin and a PD-l inhibitor could enhance antitumor potency in ESCC (**Figure 7**).

## Data Availability Statement

The raw data supporting the conclusions of this article will be made available by the authors, without undue reservation.

## Ethics Statement

The animal study was reviewed and approved by Ethics Commission of the First Affiliated Hospital of Zhengzhou University.

## Author Contributions

Experiment design: FW, YL, and SZ. Database analysis: YaY, MX, and AW-G. Vitro studies: DX, YL, LG, and YuY. Vivo studies: DX, YL, and YC. Article writing: YL and DX. All authors contributed to the article and approved the submitted version.

## Funding

This work was supported by the National Natural Science Funds of China (No. 81672442), Henan Provincial Science and Technology Research Project (No. SBGJ202002080, No. JBKY202102), Leading Talent Training in Henan Province (No. YXKC2020017), and Key Scientific Research Project of Henan Universities (21A320040).

## Conflict of Interest

The authors declare that the research was conducted in the absence of any commercial or financial relationships that could be construed as a potential conflict of interest.

## Publisher’s Note

All claims expressed in this article are solely those of the authors and do not necessarily represent those of their affiliated organizations, or those of the publisher, the editors and the reviewers. Any product that may be evaluated in this article, or claim that may be made by its manufacturer, is not guaranteed or endorsed by the publisher.
