# Erratum: Metformin Downregulates PD-L1 Expression in Esophageal Squamous Cell Carcinoma by Inhibiting IL-6 Signaling Pathway

**DOI:** 10.3389/fonc.2021.832786

**Published:** 2021-12-29

**Authors:** Frontiers Production Office

**Affiliations:** Frontiers Media SA, Lausanne, Switzerland

**Keywords:** metformin, PD-L1, anti-PD-1 antibody, esophageal squamous cell carcinoma, IL-6/JAK2/STAT3 signaling pathway

Due to a production error, the title was incorrectly written as “Metformin Downregulates PD-L1 Expression in Esophageal Squamous Cell Catrcinoma by Inhibiting IL-6 Signaling Pathway”. The correct title is “Metformin Downregulates PD-L1 Expression in Esophageal Squamous Cell Carcinoma by Inhibiting IL-6 Signaling Pathway”.

The publisher apologizes for this mistake.

The original version of this article has been updated.

