# Supplementary material for: Metformin Downregulates PD-L1 Expression in Esophageal Squamous Cell Carcinoma by Inhibiting IL-6 Signaling Pathway
Source: Front Oncol. 2021 Nov 22;11:762523. doi: 10.3389/fonc.2021.762523 (PMC8645640; doi:10.3389/fonc.2021.762523)
Supplement: Supplementary file 1 [file DataSheet_1.docx]

Metformin downregulates PD-L1 expression in esophageal squamous cell carcinoma by inhibiting IL-6 signaling pathway

Supplementary Material

**Supplementary Table 1. The design of shRNA targeting JAK2**

| **NO.** | **Accession** | **Target Seq** | **Titer** |
| --- | --- | --- | --- |
| JAK2-RNAi(80059-1) | NM_004972 | TAGCTCATTAAGGGAAGCTTT | 2E+9 |
| JAK2-RNAi(80060-1) | NM_004972 | AAGCAACTGTCATGGCCCAAT | 2E+9 |
| JAK2-RNAi(80061-1) | NM_004972 | CTGCAGTACACATCTCAGATA | 2E+9 |

#
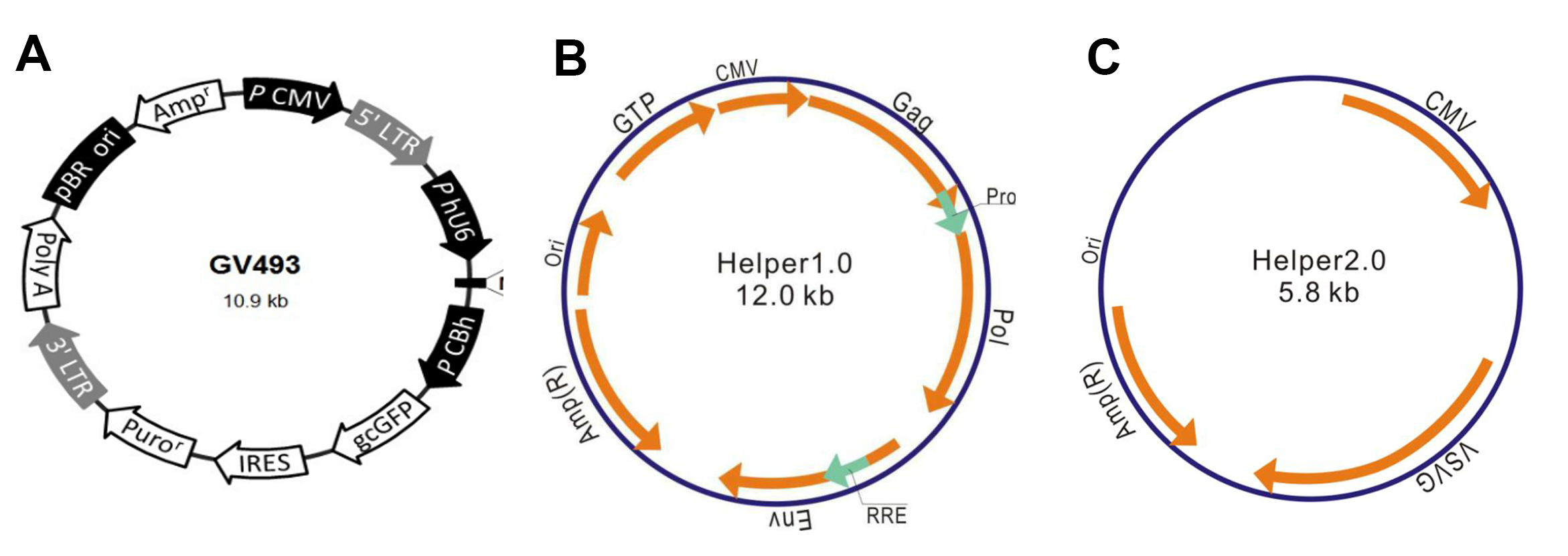


**Supplementary Figure 1.** The schematic diagram of viral vector. (A) GV493; (B) pHelper 1.0; (C) pHelper 2.0.


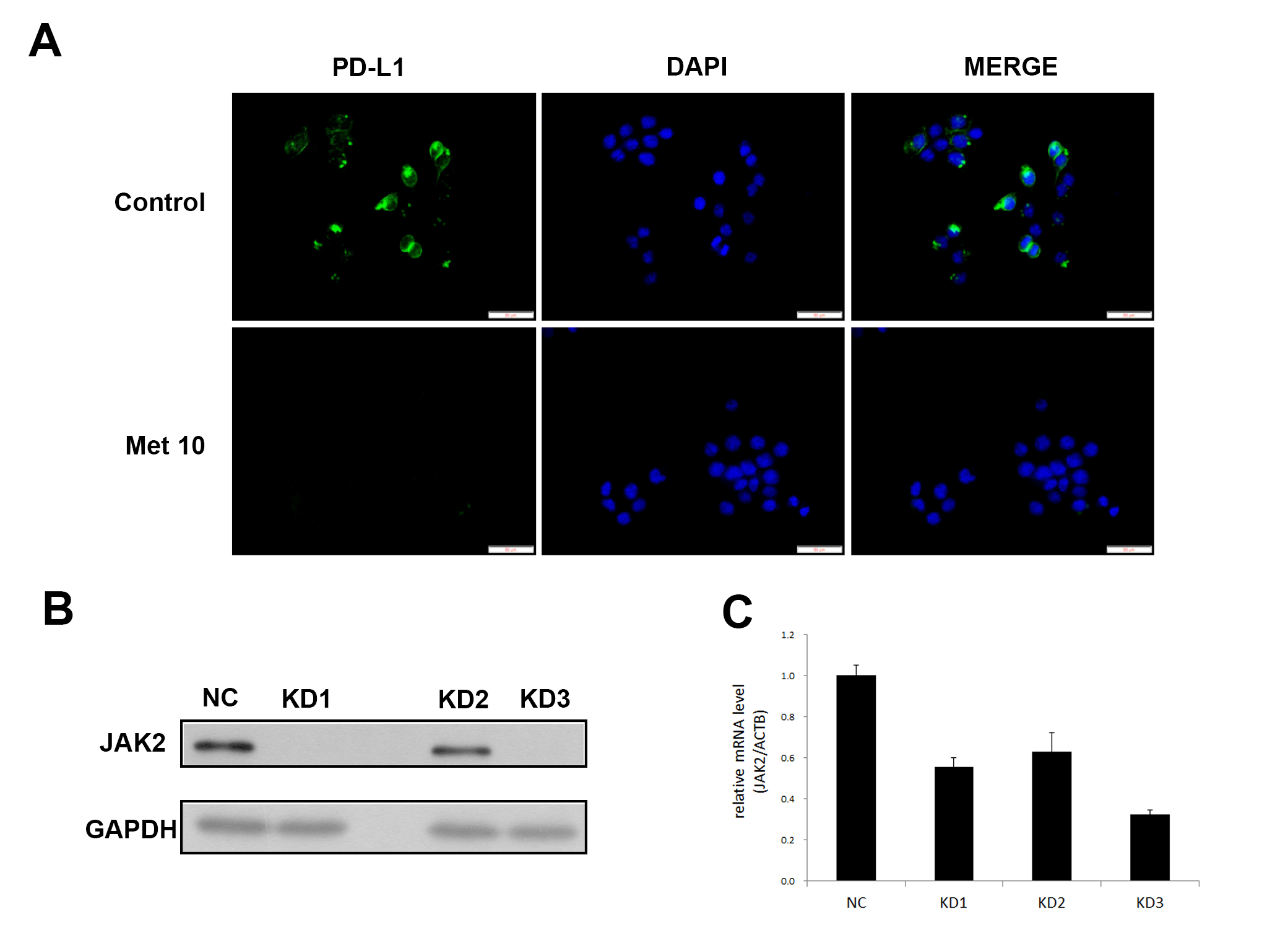


**Supplementary Figure 2.** A. Immunofluorescence assay showed that the expression of PD-L1 was decreased in KYSE-450 cells treated with 10mM metformin. B. Western blot analysis of JAK2 in KYSE450 cells transfected with three JAK2 knockdown plasmid. C. mRNA expression levels of JAK2 in KYSE450 cells transfected with three JAK2 knockdown plasmid. Finally, we selected KD3 plasmid for subsequent experiments.
